# *In vitro* Mechanistic Exploration of Novel Spiropyrrolidine Heterocyclic Hybrids as Anticancer Agents

**DOI:** 10.3389/fchem.2020.00465

**Published:** 2020-06-03

**Authors:** Raju Suresh Kumar, Abdulrahman I. Almansour, Natarajan Arumugam, Faruq Mohammad, Raju Ranjith Kumar

**Affiliations:** ^1^Department of Chemistry, College of Science, King Saud University, Riyadh, Saudi Arabia; ^2^Department of Organic Chemistry, School of Chemistry, Madurai Kamaraj University, Madurai, India

**Keywords:** molecular biology, *in vitro* studies, role of caspases, spiropyrrolidine hybrid heterocycles, *N*-methylation

## Abstract

Novel spiro acenaphthylene pyrrolo[1,2-*b*]isoquinoline/pyrrolidine hybrids have been achieved through Pictet-Spengler/Eschweiler-Clarke reactions depending on the substitution in the benzyl ring. The *in vitro* biological efficacy of *N*-methyl spiropyrrolidine derivatives toward different cancer and non-cancer cell lines revealed that these novel spiro heterocyclic hybrids induced cancer cell death at moderate concentrations, while slight reduction in non-cancer cell viability at the higher concentrations. The analysis of cancer cells proved that the major pathway of cell death is apoptosis and in addition, the role of caspases is confirmed by the appearance of fluorescent cells in microscopic images. Therefore, this study indicates a sustainable way of treating cancer cells by inducing apoptotic pathways and associated caspases, while simultaneously protecting the non-cancer cells.

## Introduction

Cancer is a genetic disorder and its occurrence to the healthy normal tissues causes an abnormal growth called the tumors and further the affected cells lose their regular functioning, ability to grow, and division. The cancer causing genetic changes can either be inherited from the parents, lifestyle (food, daily life, geographical region, etc.), or environmental exposures (tobacco smoke, toxic chemicals, UV radiation, etc.) and can result in the damage of DNA. The extent of genetic disorder can be different even for similar types of cells although they live within the same tissue and in addition, the response of normal cell can be different toward the genetic changes than the corresponding cancer cells. Similarly, the healthy normal cells are mostly immune toward some therapeutic drugs and this resistant rate to the normal cells is quite high as compared against the cancer diseased cells (Cattley and Radinsky, 2004; Horsman and Vaupel, 2016).

The major drawback of traditional chemotherapeutic agents is that they also affect the healthy normal cells during the cancer treatment. However, the extent of cell death can be different among the cancer and non-cancer cells when get exposed to the same therapeutic drug as the operating mechanism is different. Among many different drugs which fall into this category, spiro heterocycles embedded with pharmacologically active units are encouraging applicants for drug discovery since they are likely to interact more proficiently with binding pockets in proteins, which are three-dimensional in nature, and have better solubility, a key property in the course of drug development (Zheng et al., 2014). The medicinal activities of these heterocycles were due to the presence of different structural moieties, and these compounds have the capacity to interfere with the biological systems in a sequential way of protein inhibition or enzymatic degradation pathway. The basic mechanism of these compounds being the induction of apoptosis by binding to the DNA and also acting against the cell division and growth with the arrest of cell cycle, where all these pathways leads to the cancer cell death. Furthermore, the spiro heterocyclic hybrids also serves as the effective inhibitors of many different proteins that includes SIRT1 (Heltweg et al., 2006; Milne and Denu, 2008; Balcerczyk and Pirola, 2010; Rambabu et al., 2013), Mdm2-p53 (Ding et al., 2005; Zhao et al., 2013; Rew et al., 2014; Wang et al., 2014), PLK4 etc and thereby showing affinity for the anaplastic lymphoma kinase (ALK) receptor (Benabdallah et al., 2018). Therefore, taking advantage of the physiological behavior of cancer and non-cancer cell's response toward therapeutics, the current study is aimed to develop some novel spiro hybrid heterocycles possessing drug agents that are capable of inducing the cell death pathways to the cancer cells in a controllable manner.

Recent progress on spiro building blocks facilitates incorporation of spiro scaffolds into more pharmaceutically active molecules. The spiro heterocyclic hybrids besides possessing three-dimensionality than flat aromatic compounds, also introduce structural novelty. Nevertheless, both spiro and flat aromatic compounds can impact ligand binding entropy, it has been recommended that compounds with more flat rings have suboptimal physical properties and are less likely to be successfully developed as drugs (Lovering et al., 2009; Richie and Macdonald, 2009; Lovering, 2013; Aldeghi et al., 2014). As a consequence, in recent literatures, the spiro heterocyclic hybrids have appeared progressively (Marson, 2011).

In addition, synthesis of hybrid heterocycles possessing two or more diverse biologically active structural units in a single molecular framework has been viewed as one of the most treasured protocol in drug development so as to obtain novel therapeutic approaches to treat cancer diseases (Viegas-Junior et al., 2007; Nepali et al., 2014; Kerru et al., 2017; Nekkanti et al., 2017; Cascioferro et al., 2020). The design and development of anticancer agents comprising two or more biologically active structural units with antitumor activity headed to the evolution of molecules with enhanced activity profile compared to the parent compounds. It is also pertinent to note that hybrid anticancer agents displayed better specificity, a superior aptitude to overcome drug-resistance mechanisms, better patient compliance and reduced side effects (Yang and Fu, 2015).

Prompted by these reports, we explore in the present investigation the synthesis of some novel spiro heterocyclic hybrids via Pictet-Spengler or Eschweiler-Clarke reactions depending on the substitution in the benzyl ring of the azomethine ylide. The azomethine ylide with no substitution on the aryl ring proceeded via Pictet-Spengler route to furnish the spiro acenaphthylene pyrrolo[1,2-*b*]isoquinoline hybrids as anticipated, while the azomethine ylide substituted with 4-OH group progressed via Eschweiler-Clarke route affording the unpredicted *N*-methylated spiro acenaphthylene-pyrrolidine heterocyclic hybrids. Subsequently, these derivatives were tested toward their biological efficiency by means of *in vitro* cell culture assays. For the *in vitro* studies, the derivatives were first tested for the cell viability and proliferation in the presence of different cancer and non-cancer cells and further the active role played by the apoptosis pathway to bring the cancer cell to death was also studied.

## Materials and Methods

### Chemistry

The details of materials and methods employed in the present work has been provided in the Supplementary Data section and is similar to our earlier publications (Kumar et al., 2018).

#### General Procedure for the Synthesis of Spiro[acenaphthene-2′.2-pyrrolidin]-1′-ones 5(a–f)

Paraformaldehyde (1 mmol) was added to a solution of **4** (1 mmol) in 10 mL of dicholoromethane followed by trifluoroacetic acid (0.1 mmol). The reaction mixture was stirred overnight. After completion of the reaction, the stirred solution was washed with water and dried over Na_2_SO_4_. The crude product obtained was purified by column chromatography with hexane–ethyl acetate (3:2 v/v) as eluent.

#### Characterization Data for Spiro[acenaphthene-2′.2-pyrrolidin]-1′-one (5a)

Pale yellow solid; 74% yield; mp 135–137°C; IR (KBr) ν_max_ 3,356, 1,711, 1,535, 1,352 cm^−1^; ^1^H NMR (500 MHz, CDCl_3_): δ_H_ 1.87 (3H, s, N-CH_3_), 2.88 (1H, dd, *J* = 14.5, 6.0 Hz, 6-CH_2_), 2.99 (1H, dd, *J* = 14.5, 7.0 Hz, 6-CH_2_), 4.35–4.40 (1H, m, H-5), 4.62 (1H, d, *J* = 11.0 Hz, H-3), 6.42 (1H, t, *J* = 9.5 Hz, H-4), 6.75–6.91 (6H, m, ArH), 7.19 (2H, d, *J* = 8.5 Hz, ArH), 7.52–7.58 (2H, m, ArH), 7.79–7.98 (5H, m, ArH). ^13^C NMR (125 MHz, CDCl_3_): δ_C_ 35.36, 36.69, 56.06, 64.71, 80.42, 87.72, 115.36, 120.41, 120.58, 125.45, 127.47, 127.73, 127.82, 128.08, 128.15, 128.84, 129.25, 130.17, 130.65, 131.82, 133.45, 135.34, 142.86, 154.39, 208.03; LC/MS(ESI): *m/z* = 464 (M^+^); Anal. calcd for C_29_H_24_N_2_O_4_: C, 74.98; H, 5.21; N, 6.03%; found: C, 74.85; H, 5.32; N, 6.16%.

#### Characterization Data for Spiro[acenaphthene-2′.2-pyrrolidin]-1′-one (5b)

Pale yellow solid; 72% yield; mp 152–154°C; IR (KBr) ν_max_ 3,349, 1,713, 1,546, 1,358 cm^−1^; ^1^H NMR (500 MHz, CDCl_3_): δ_H_ 1.85 (3H, s, N-CH_3_), 2.86 (1H, dd, *J* = 14.5, 6.5 Hz, 6-CH_2_), 2.96 (1H, dd, *J* = 14.5, 6.5 Hz, 6-CH_2_), 4.31–4.36 (1H, m, H-5), 4.55 (1H, d, *J* = 11.0 Hz, H-3), 6.35 (1H, dd, *J* = 10.0, 9.5 Hz, H-4), 6.68 (2H, d, *J* = 9.0 Hz, ArH), 6.79 (2H, d, *J* = 8.5 Hz, ArH), 7.01 (2H, d, *J* = 8.5 Hz, ArH), 7.18 (2H, d, *J* = 9.0 Hz, ArH), 7.56–7.62 (2H, m, Ar-H), 7.81–7.88 (3H, m, Ar-H), 8.01 (1H, d, *J* = 7.5 Hz, ArH). ^13^C NMR (125 MHz, CDCl_3_): δ_C_ 35.26, 36.63, 55.42, 64.52, 80.10, 87.63, 115.35, 120.53, 120.67, 125.64, 127.48, 128.30, 128.85, 129.15, 128.43, 130.23, 130.68, 131.08, 131.38, 132.08, 133.45, 135.33, 142.85, 154.37, 207.94; LC/MS(ESI): *m/z* = 543 (M^+^); Anal. calcd for C_29_H_23_BrN_2_O_4_: C, 64.10; H, 4.27; N, 5.16%; found: C, 64.32; H, 4.11; N, 5.27%.

#### Characterization Data for Spiro[acenaphthene-2′.2-pyrrolidin]-1′-one (5c)

Pale yellow solid; 80% yield; mp 110–112°C; IR (KBr) ν_max_ 3,450, 1,712, 1,538, 1,355 cm^−1^; ^1^H NMR (500 MHz, CDCl_3_): δ_H_ 1.86 (3H, s, N-CH_3_), 2.87 (1H, dd, *J* = 14.5, 6.5 Hz, 6-CH_2_), 2.97 (1H, dd, *J* = 14.5, 7.0 Hz, 6-CH_2_), 4.32–4.38 (1H, m, H-5), 4.58 (1H, d, *J* = 10.5 Hz, H-3), 6.34–6.38 (1H, m, H-4), 6.74 (2H, d, *J* = 8.5 Hz, ArH), 6.80 (2H, d, *J* = 8.5 Hz, ArH), 6.85 (2H, d, *J* = 8.5 Hz, ArH), 7.18 (2H, d, *J* = 8.5 Hz, ArH), 7.55–7.58 (1H, m, ArH), 7.61 (1H, d, *J* = 7.5 Hz, ArH), 7.80–7.88 (3H, m, ArH), 8.00 (1H, d, *J* = 8.0 Hz, ArH). ^13^C NMR (125 MHz, CDCl_3_): δ_C_ 35.27, 36.61, 55.38, 64.55, 80.20, 87.68, 115.38, 120.57, 120.67, 125.64, 127.47, 128.27, 128.41, 128.86, 129.02, 129.08, 130.21, 130.65, 132.11, 133.45, 133.79, 135.37, 142.84, 154.47, 208.08; LC/MS(ESI): *m/z* = 498 (M^+^); Anal. calcd for C_29_H_23_ClN_2_O_4_: C, 69.81; H, 4.65; N, 5.61%; found: C, 69.65; H, 4.79; N, 5.50%.

#### Characterization Data for Spiro[acenaphthene-2′.2-pyrrolidin]-1′-one (5d)

Yellow solid; 75% yield; mp 123–125°C; IR (KBr) ν_max_ 3,542, 1,714, 1,537, 1,351 cm^−1^; ^1^H NMR (500 MHz, CDCl_3_): δ_H_ 1.86 (3H, s, N-CH_3_), 2.02 (3H, s, CH_3_), 2.87 (1H, dd, *J* = 14.0, 6.0 Hz, 6-CH_2_), 2.98 (1H, dd, *J* = 14.0, 7.5 Hz, 6-CH_2_), 4.32–4.38 (1H, m, H-5), 4.59 (1H, d, *J* = 10.5 Hz, H-3), 6.39 (1H, t, *J* = 9.5 Hz, H-4), 6.65–6.72 (4H, m, ArH), 6.80 (2H, d, *J* = 8.0 Hz, ArH), 7.19 (2H, d, *J* = 8.5 Hz, ArH), 7.52–7.55 (1H, m, ArH), 7.59 (1H, d, *J* = 6.5 Hz, ArH), 7.77–7.90 (3H, m, ArH), 7.97 (1H, d, *J* = 8.0 Hz, ArH). ^13^C NMR (125 MHz, CDCl_3_): δ_C_ 20.76, 35.30, 36.67, 55.81, 64.68, 80.34, 88.06, 115.35, 120.42, 120.55, 125.37, 127.62, 128.07, 128.83, 128.89, 129.30, 130.18, 130.62, 131.65, 131.81, 133.40, 135.34, 137.46, 142.91, 154.39, 208.25; LC/MS(ESI): *m/z* = 478 (M^+^); Anal. calcd for C_30_H_26_N_2_O_4_: C, 75.30; H, 5.48; N, 5.85%; found: C, 75.48; H, 5.35; N, 5.74%.

#### Characterization Data for Spiro[acenaphthene-2′.2-pyrrolidin]-1′-one (5e)

Yellow solid; 69% yield; mp 116–118°C; IR (KBr) ν_max_ 3,339, 1,712, 1,540, 1,353 cm^−1^; ^1^H NMR (500 MHz, CDCl_3_): δ_H_ 1.85 (3H, s, N-CH_3_), 2.87 (1H, dd, *J* = 13.5, 6.5 Hz, 6-CH_2_), 2.98 (1H, dd, *J* = 13.5, 4.0 Hz, 6-CH_2_), 3.81 (3H, s, OCH_3_), 4.32–4.38 (1H, m, H-5), 4.57 (1H, d, *J* = 11.5 Hz, H-3), 6.35 (1H, dd, *J* = 10.0, 9.5 Hz, H-4), 6.77 (2H, d, *J* = 8.0 Hz, ArH), 6.83 (2H, d, *J* = 8.5 Hz, ArH), 6.89 (2H, d, *J* = 8.5 Hz, ArH), 7.02 (2H, d, *J* = 9.0 Hz, ArH), 7.53–7.62 (2H, m, ArH), 7.76–7.80 (3H, m, ArH), 7.98 (1H, d, *J* = 8.0 Hz, ArH). ^13^C NMR (125 MHz, CDCl_3_): δ_C_ 35.38, 36.61, 54.94, 55.06, 64.67, 80.34, 88.09, 113.59, 115.34, 120.53, 120.78, 125.42, 126.94, 128.12, 128.87, 129.68, 130.29, 130.65, 131.09, 131.18, 131.76, 134.69, 142.75, 154.42, 160.55, 207.75; LC/MS(ESI): *m/z* = 494 (M^+^); Anal. calcd for C_30_H_26_N_2_O_5_: C, 72.86; H, 5.30; N, 5.66%; found: C, 72.70; H, 5.41; N, 5.79%.

#### Characterization Data for Spiro[acenaphthene-2′.2-pyrrolidin]-1′-one (5f)

Yellow solid; 78% yield; mp 140–142°C; IR (KBr) ν_max_ 3,359, 1,712, 1,535, 1,356 cm^−1^; ^1^H NMR (500 MHz, CDCl_3_): δ_H_ 1.89 (3H, s, N-CH_3_), 2.90 (1H, dd, *J* = 14.0, 6.0 Hz, 6-CH_2_), 2.98 (1H, dd, *J* = 14.0, 7.5 Hz, 6-CH_2_), 4.36–4.41 (1H, m, H-5), 4.67 (1H, d, *J* = 11.0 Hz, H-3), 6.44 (1H, dd, *J* = 10.0, 9.5 Hz, H-4), 6.81 (2H, d, *J* = 8.0 Hz, ArH), 7.11–7.21 (4H, m, ArH), 7.53–7.60 (3H, m, ArH), 7.79–7.85 (2H, m, ArH), 7.90 (1H, d, *J* = 8.0 Hz, ArH), 7.93 (1H, d, *J* = 7.0 Hz, ArH), 8.00 (1H, d, *J* = 8.0 Hz, ArH). ^13^C NMR (125 MHz, CDCl_3_): δ_C_ 35.28, 36.55, 55.31, 64.45, 80.12, 87.11, 115.39, 120.77, 122.97, 123.06, 126.00, 128.33, 128.93, 129.06, 129.30, 130.28, 130.80, 131.16, 132.32, 133.35, 134.46, 134.72, 136.37, 142.76, 147.71, 154.41, 207.62; LC/MS(ESI): *m/z* = 509 (M^+^); Anal. calcd for C_29_H_23_N_3_O_6_: C, 68.36; H, 4.55; N, 8.25%; found: C, 68.54; H, 4.38; N, 8.37%.

### Molecular Biology Testings

In order to study the molecular biology effects of synthesized spiropyrrolidine heterocyclic hybrids, we have used the *in vitro* toxicology tests and for that, two different cell types, each of cancer and non-cancer origin were employed. The non-cancer cell lines include the L929 mouse fibroblasts and MCF10 breast cells, while the cancer cell lines are A549 human alveolar basal epithelial cells and Jurkat human T lymphocyte cells. Following the MTT assay induced toxicology studies at two different time periods of 24 and 48 h, the IC_50_ values (minimum concentration required for 50% of cells to loss the viability) were determined. Further, the influence of apoptosis and caspase activity were investigated by selecting only the A549 cells at their IC_50_ concentration as these cells were found to be the most significant in terms of inducing the toxicity during the MTT assay as against the Jurkat and other non-cancer cells. All the biological studies were carried by selecting Camptothecin (CPT) as a positive control and the cells of no treatment as the negative control. The reason for selecting this particular compound as a positive control is that it is a well-known anticancer agent and acts by the mechanism of topoisomerase I inhibition along with the DNA cleavage, subsequent inhibition of litigation, and finally leads to DNA strand breaks. The detailed procedures for the cell culturing and assay protocols were mentioned elaborately in our earlier publications (Bwatanglang et al., 2016a,b; Kumar et al., 2018). Each experiment was repeated thrice and the results expressed as the mean ± standard deviation of all the data values. From the graph, the ^*^ and ^**^ corresponds to *p* < 0.05 and *p* < 0.01 vs. the untreated-control measurements.

## Results and Discussion

**Scheme 1:**
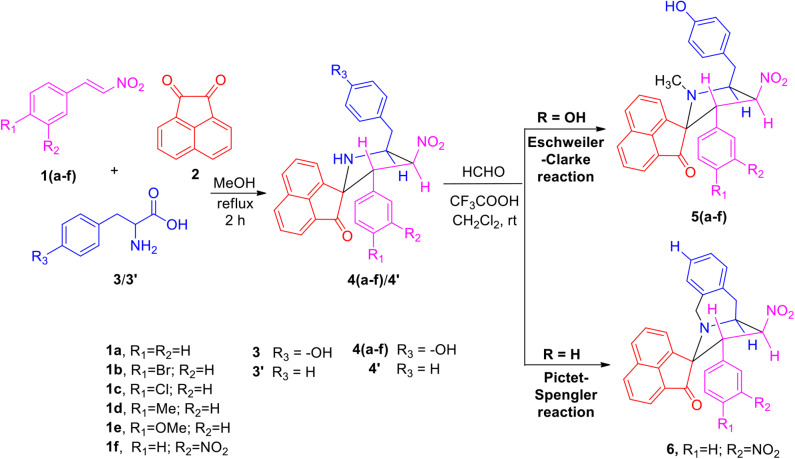
Synthesis of *N*-methyl spiropyrrolidine heterocyclic hybrids **5(a–f)**.

The precursors **4(a–f)** and **4′** were synthesized employing a three-component 1,3-dipolar cycloaddition reaction of β-nitrostyrenes **1(a**–**f)**, acenaphthenequinone **2** and tyrosine **3** or phenylalanine **3′** (Scheme 1). It is noteworthy that the azomethine ylide derived from the combination of acenaphthenequinone and tyrosine has not been explored much in the literature. In a typical 1,3-dipolar cycloaddition reaction, an equimolar amount of **1**, **2** and **3** was refluxed in methanol for 2 h (Kumar et al., 2018) and after completion of the reaction, the crude spiroheterocyclic hybrids **4(a–f)/4′** obtained were purified by column chromatography and was employed for further reactions.

As these spiropyrrolidine heterocyclic hybrids **4(a–f)** possesses a benzyl sub-unit next to the amino group (-NH) of the pyrrolidine ring, these compounds were subjected to Pictet-Spengler cyclization. In a representative reaction, the spiropyrrolidine **4f** (1 mmol) was dissolved in 10 mL of CH_2_Cl_2_ followed by paraformaldehyde (1 mmol) and trifluoroacetic acid (0.1 mmol), the reaction mixture being stirred at ambient temperature overnight. After the reaction is completed as obvious from TLC, the reaction mixture was washed with water and dried over Na_2_SO_4_. Column chromatography was performed with hexane–ethyl acetate (3:2 v/v) to obtain the pure product. Spectral characterization of the obtained product (*vide* supporting data) discovered the creation of *N*-methylated spiroheterocyclic hybrid **5f** through Eschweiler-Clarke reaction. The expected Pictet-Spengler cyclization was not observed. Similarly, all other spiroheterocyclic hybrids **4(a–e)** derived from different substituted nitrostyrenes **1(a–e)** when subjected to the same reaction conditions also afforded only the *N*-methylated spiroheterocyclic hybrids **5(a–e)**, the pictet-Spengler product was not witnessed in all these reactions even in traces.

In order to account for the formation of *N*-methylated product, we also attempted the synthesis of spiro compounds employing the starting substrate with unsubstituted benzyl sub-unit viz. spiro-pyrrolo-acenaphthene hybrid **4′** under the same reaction conditions employed before. The anticipated Pictet-Spengler cyclization product **6** was attained in low yield along with some uncharacterizable impurities. The absence of formation of Eschweiler-Clarke product revealed that the substitution in the benzyl sub-unit of the spiroheterocyclic hybrid **4** supports the *N*-methylation and plays an important role in the development of Eschweiler-Clarke product.

**Scheme 2:**
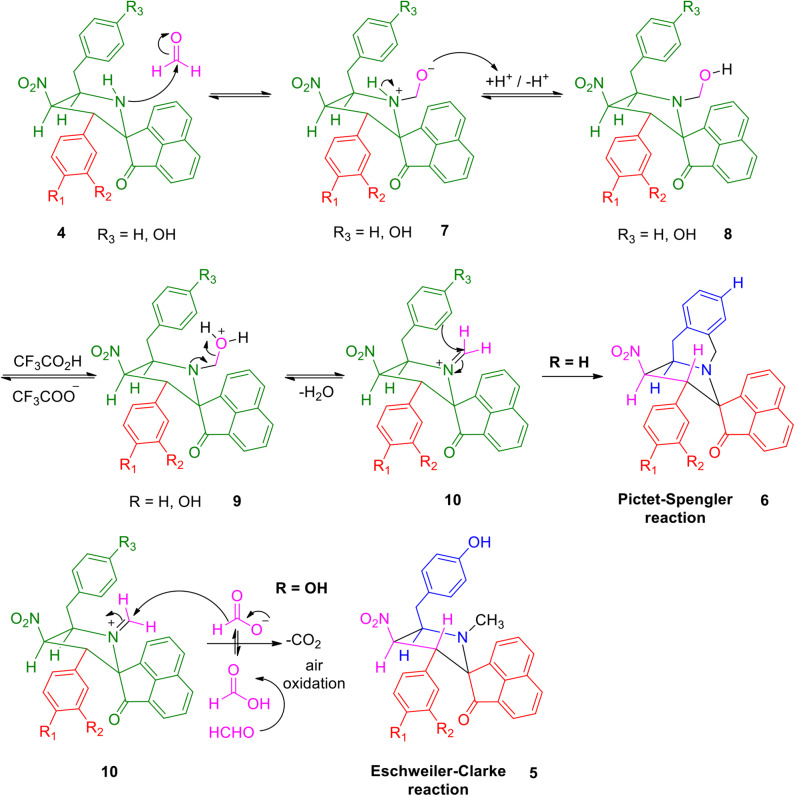
Feasible mechanism for the construction of spiroheterocyclic hybrids **5** and **6**.

A possible validation for the construction of spiroheterocyclic hybrids **5** and **6** is appended in Scheme 2. Initially, the carbonyl group of formaldehyde is being attacked by -NH group of the spiropyrrolidine furnishing intermediate **7** which then rearranges to the alcohol **8**. The iminium ion **10** has been formed from the alcohol **8** via compound **9**. Further reaction of the intermediate **10** is being determined by the substituent (H/OH) present in the benzyl subunit. When the para position of the benzyl subunit is unsubstituted (R_3_=H), Pictet-Spengler product **6** is favored, involving the cyclization of the iminium carbon of the pyrrole ring with the meta-carbon (with respect to substituent R_3_) of the benzyl group. When the para position of the benzyl group is substituted with –OH (R_3_=OH), the Pictet-Spengler product is not favored since the mesomeric effect induced by *p*-OH group diminishes the electron density on the meta-carbon of the benzyl group. As a consequence, the iminium ion **10** with *p*-OH group leads to the formation of *N*-methylated spiro-heterocyclic hybrids **5** via Eschweiler-Clarke reaction through a reaction sequence which involves, the air oxidation of formaldehyde to formic acid, which subsequently loses CO_2_ to act as a hydride donor which then reduces the iminium ion **10** to furnish the *N*-methylated spiroheterocyclic hybrids **5**.

### Molecular Biology Studies

**Figure 1:**
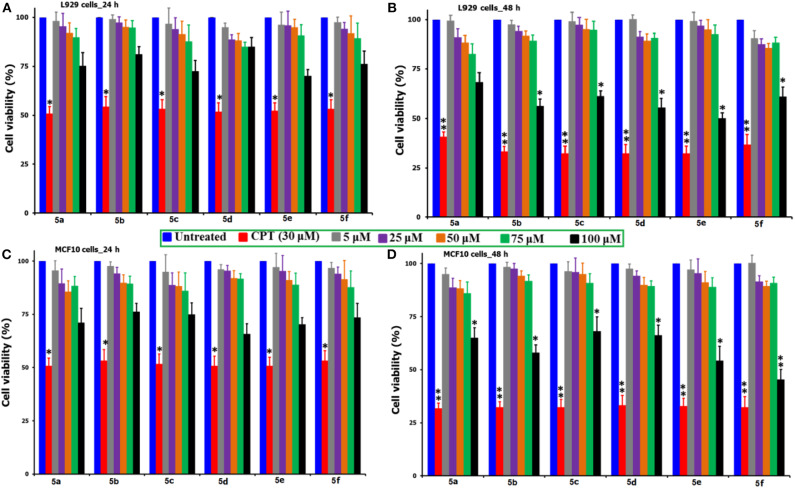
Comparison of the % cell viability studies of **5(a–f)** as against the positive control of CPT (30 μM) followed by the exposure to non-cancer cell lines of L929 mouse fibroblast and MCF10 breast cells over two different time periods of 24 and 48 h. *Corresponds to the significant and **corresponds to the highly significant values. From the figure, the incubation of L929 cells shown as **(A)** 24 h and **(B)** 48 h, while the MCF10 cells as **(C)** 24 h and **(D)** 48 h.

In order to test the behavior of the synthesized *N*-methylated spiro heterocyclic hybrids **5(a-f)** toward the non-cancer cells, two different cell lines, L929 mouse fibroblast cells and MCF10 breast cells were selected. Figure 1 compares the MTT-induced cell viability and proliferation studies following the exposure of compounds **5(a-f)** up to 100 μM concentration and over the incubation periods of 24 and 48 h, where a positive control of CPT (30 μM) was used. From the figure, it can be observed that there is no significant loss to the viability of cells up to the 75 μM concentration for both of the cell types and for all of the tested compounds, while for the CPT under the tested concentration of 30 μM, we observed near to a 50% mortality. For our synthesized compounds, we were able to observe a significant loss in the cell viability only at the highest tested concentration of 100 μM (48 h) and this provides the preliminary information that the direct exposure of cells to this concentration of above be only lethal. What particularly inferred from this study is that our synthesized compounds are not so aggressive toward the non-cancer cells and there lies some safety zone where the non-targeted healthy cells can be protected from the highly toxic chemotherapeutic agents. That too at the highest tested concentration of 100 μM for our synthesized compounds, the observed % of cell viability losses are far lower as compared with that of CPT (30 μM) and this provides a hidden message that these healthy normal cells are immune to the toxic induced responses due to the strong intracellular physiological pathways. Further, the compounds are said to be efficient only when they show significant cell loss toward the cancer cells at the lesser concentrations than the 100 μM and before the 48 h incubation period.

**Figure 2:**
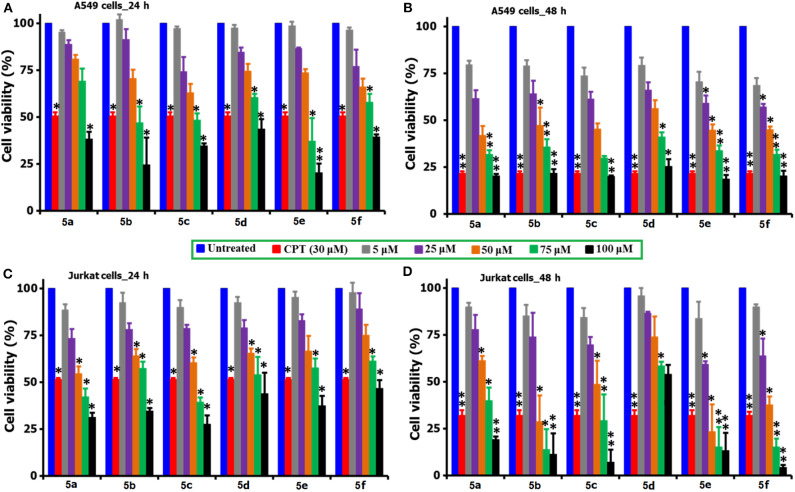
Comparison of the cell viability and proliferation studies of **5(a–f)** treated A549 and Jurkat cell cultures over 24 and 48 h periods of time. For the studies, a positive control of CPT (30 μM) was selected and the cells of no treatment as negative controls. *for significance and **for highly significance values. From the figure, the incubation of A549 cells shown as **(A)** 24 h and **(B)** 48 h, while the Jurkat cells as **(C)** 24 h and **(D)** 48 h.

The MTT reagent-induced *in vitro* cell viability and proliferation studies following the exposure of compounds **5(a-f)** to the two cancer cell lines (A549 and Jurkat) over two different time periods of 24 and 48 h are compared and shown in Figures 2A–D. Similarly, Table 1 describes the IC_50_ values of the same compounds **5(a-f)** over the two cancer cell lines and time periods. From the analysis of results shown in Figure 2 and Table 1, one can identify that the tested compounds as compared to the positive (CPT) and negative controls are affecting the cell viability significantly. Also, the loss of viability seems to be increased with an increase in the concentration and incubation period and this provides the preliminary information that the tested compounds are maintaining some levels of therapeutic behavior toward the cancer cells and if it can be guided carefully, can lead to the anticancer activity. Surprisingly, the two cancer cells are reacting differently to the tested compounds, i.e., the A549 cells seem to be more sensitive to the tested compounds during the first 24 h of exposure period, however the Jurkat cells becoming more responsive during the 48 period of exposure. This difference in the sensitivity of two cancer cells to the same testing compound can be attributed to the changes in the resistive response of each cell types which is supported by the intracellular protein mechanisms (Mohammad et al., 2014; Bwatanglang et al., 2017). Since the amount of intracellular proteins secreted by the cells are unique to each individual cell type and so in that way some cells may react very fast as compared to the others. Also, the efficiency of any chemotherapeutic agent is decided by its ability to reduce the cell count at the earliest possible time and in that way we have identified the A549 cells to be the highly responsive cell type to the compound **5e** over a 24 h period as compared to the other compounds, cells, and time periods. The visual observation of cells by means of microscopic observation also be confirming for a decrease in the number of cells for the compound **5e** treated cells as against the positive control of CPT (30 μM) and negative control of without any treatment (Figures S11, S11a). We observed the least IC_50_ value of 55.25 ± 2.15 μM for the compound **5e** during the 24 h exposure period for the A549 cell line, while at the 48 h of exposure, a IC_50_ value of 20.52 ± 3.6 μM was observed for the same compound. In a general way, our interest is with the therapeutic agent of higher efficiency at the shortest exposure period and so we carried the following mechanistic studies linked to the cell death for the compound **5e** at the 24 h exposure period only. Although the compound **5f** is showing the very high loss (90%) of cell viability at its highest concentration of 100 μM toward the Jurkat cells, its efficiency is considered to be low (as against **5e**) because of the fact that the compound **5f** is exhibiting such levels at the highest exposure period (48 h). However, the compound **5e** is offering almost similar effects (80% loss) even at the 24 h exposure period and so we selected to test the compound **5e** (at its IC_50_ of 55 μM) for the further analysis of cell death mechanisms.

**Table 1:** Comparison of IC_50_ values of compounds **5(a–f)** over 24 and 48 h incubation periods for A549 and Jurkat cells.

**Entry**	**Comp**	**IC**_****50****_ **μM (24 h)**		**IC**_****50****_ **μM (48 h)**	
		**A549**	**Jurkat**	**A549**	**Jurkat**
1	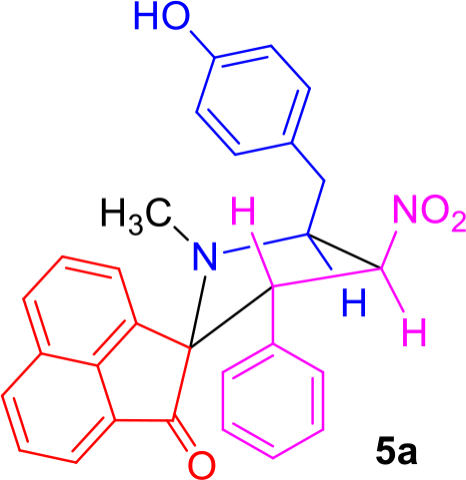	84.19 ± 4.2	65.62 ± 2.1	68.55 ± 3.1	31.45 ± 2.3
2	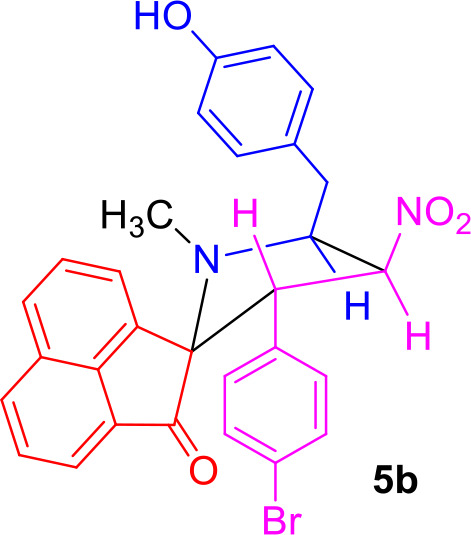	79.5 ± 3.15	82.41 ± 4.0	36.25 ± 4.5	52.24 ± 3.5
3	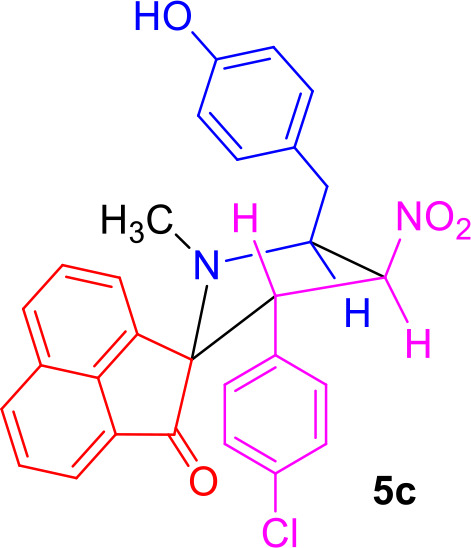	77.3 ± 4.56	63.25 ± 3.2	52.3 ± 2.2	59.45 ± 4.1
4	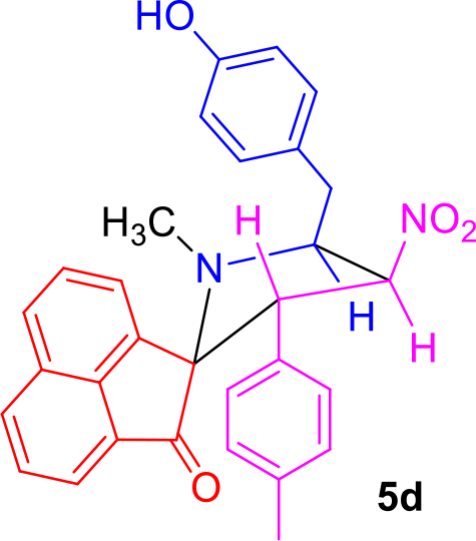	82.07 ± 2.55	78.15 ± 2.6	70.2 ± 2.8	47.45 ± 3.8
5	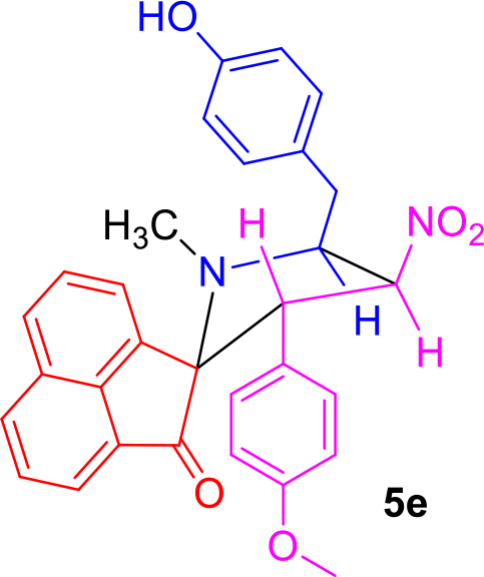	55.25 ± 2.15	81.24 ± 3.1	20.52 ± 3.6	61.25 ± 4.6
6	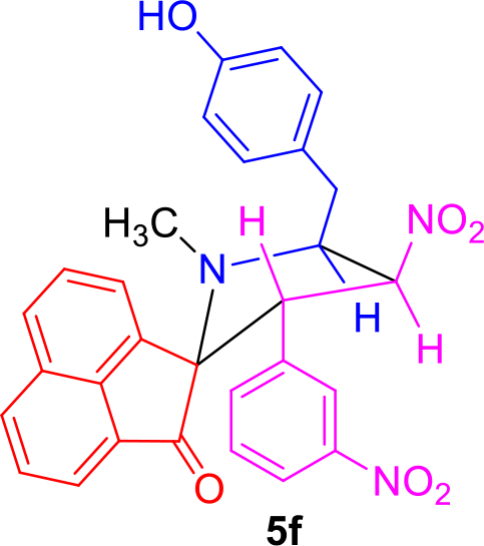	70.65 ± 3.45	92.45 ± 2.8	41.25 ± 2.4	62.47 ± 4.4

**Figure 3:**
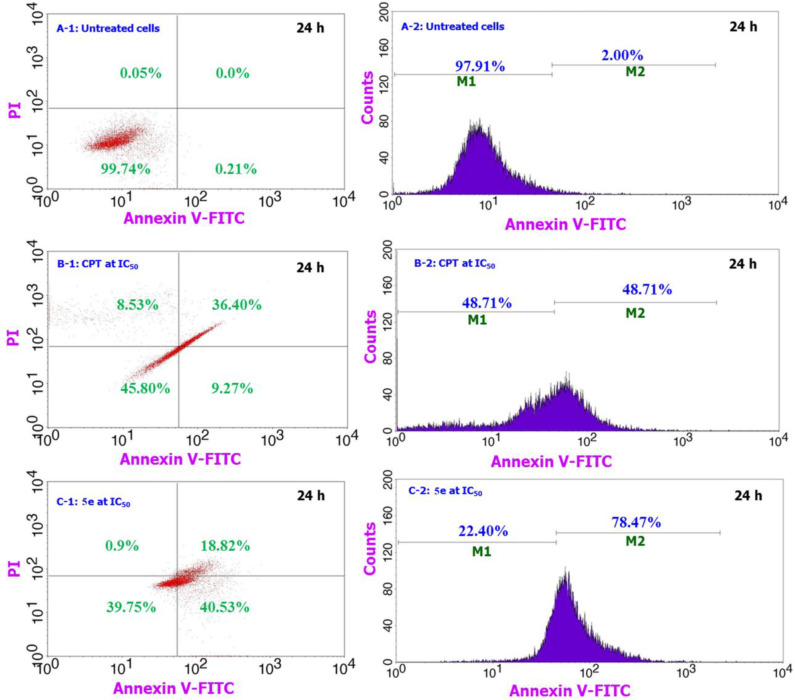
Comparison of the apoptosis assay results for **5e** treated cells along with the CPT treated and untreated cells over 24 h period for A549 cells. From the figure, **A-1** & **A-2** corresponds to the negative control, **B-1** & **B-2** for the positive control of CPT, and **C-1** & **C-2** for our testing sample 5e.

The compound **5e** induced apoptotic pathway for A549 treated cells over a 24 h period is compared against the positive (CPT, 30 μM) and negative controls and the results are shown in Figure 3. Similarly, the apoptosis study over the 48 h exposure period is shown in the supporting information of Figure S12. As compared to the behavior of cells shown in Figures 3A-1,B-1, the compound **5e** treated cells (C-1) are experiencing a significant amount of apoptotic pathway. We observed from the analysis that almost 99% cells are live and did not induce any apoptotic or necrotic pathways for the normal-untreated cultures (A-1), while the positive control (CPT) treated cells are having only 45% live cells, 9% apoptotic, and 36% late apoptotic or early necrosis pathway. These numbers for the compound **5e** treated cells are getting shifted to 39% of live cells, 40% apoptotic, and 18% late apoptotic or early necrosis pathways. The observation of only 9% apoptotic cells for the CPT treated cells while 40% for the **5e** treated cells provides a preliminary indication of the way of handling the cancer cells by the compound **5e**. Since the CPT acts directly onto the cancer cells and responsible for the accidental or irreversible pathway of reducing the cancer cell number and however, the compound **5e** works in a systematic way and induces the cancer cell death almost similar to CPT. In addition, the live and apoptotic cell count represented by M1 and M2 in the Figures 3B-2,C-2 reveals that there is significant amount of cells (about 78%) that are experiencing the apoptotic pathway for compound **5e** treated cells as against only 48% for the CPT treated ones. The number of cells experiencing the apoptotic pathway are getting increased on increasing the incubation time from 24 to 48 h (Figure S12) to 88% (**5e** treated cells) and 63% (CPT treated cells) and this indicates that the **5e** compound has the same activity against the cancer cells even after the 24 h period (up to 48 h) and during this period too, the cells are having the apoptotic cell death pathway. Further, the property of enhancing our compound treated cells number to experience the apoptotic pathway can particularly be highly useful during the cancer chemotherapeutic treatment while handling of the non-cancer cells in such cases becomes easier where the drug shows its effect to the healthy and non-targeted sites. Since the cells which are experiencing the apoptotic pathway can be reversible and in that way the healthy normal cells can be reverted to their normal stage and at the same time the cancer cells can be molded to the late apoptotic or early necrosis pathways by applying some physiological changes to the cells.

**Figure 4:**
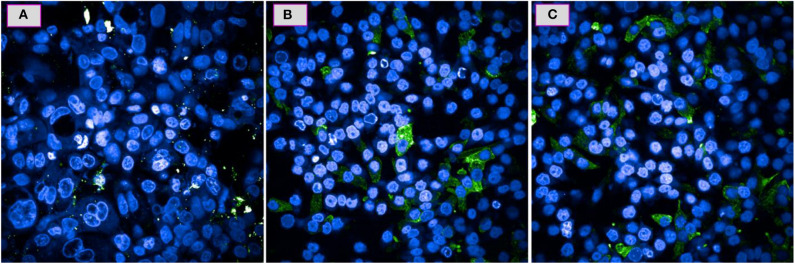
Caspase activity for the A549 cells over a 24 h period when tested at IC_50_ of **5e (C)** and the positive control of CPT **(B)** and a negative control of no treatment **(A)**.

Based on the results provided by the apoptotic assay, compound **5e** was further tested toward the role of caspases as these are the proteins which get released in response to the specific death stimuli. Figures 4A–C shows the comparison of fluorescence microscopic images of A549 cells response toward the compound **5e** against the positive control CPT and negative control of cells having no treatment. It can be observed from the Figure 4C that the **5e** treated cells are experiencing the similar behavior with that of the CPT treated ones (Figure 4B) in terms of releasing the caspases and this can be confirmed by the observation of green and blue fluorescent dye that is getting distributed in the cell's cytoplasm and nuclei, respectively. Similarly, the observation of no such green fluorescence in the cytoplasm of cells shown in Figure 4A means that there is no initiation of such pathways in that particular culture. In general, the caspase release is exhibited by the cells which are experiencing the apoptotic pathway of cell death only and the cells undergoing early necrosis or direct cell death do not follow this step. In addition, the amount of green fluorescence shown in Figure 4C is higher than the corresponding cells shown in Figure 4B and this provides further evidence for the observation of higher amount of apoptotic cells and in other words, apoptosis is the majorly drawing pathway for the compound **5e** treated cells.

Based on the cumulative analysis of results, we found that the synthesized *N*-methylated spiroheterocyclic hybrids are exhibiting the apoptotic pathway against the cancer cells and this mechanism is executed by the release of caspases. On testing the cell viability and proliferation, all the synthesized derivatives are showing some level of toxicity toward the cancer cells, with the highest being for the compound **5e**, and the observation of such high toxicity against the other compounds can be attributed to the availability of naked oxygen atom at the para position of phenyl ring. We hypothesized from the results that the phenyl ring with its methoxy group at the para position is able to effectively oxidize the intracellular proteins of the cancer cells as against other substituted groups in the spiroheterocyclic moiety. The general observation of apoptotic activity by the cancer cells due to their oxidation from the compound **5e** is considered to be a significant approach for the cancer treatment, as the tested compound **5e** along with other compounds are not really aggressive toward the non-cancer cells. We also observed on prolonged incubation times (24–48 h) that the same apoptotic pathway is getting continued which provides the consistency of compound **5e**'s mechanism against the cancer cells. This particular point of enhancing the treatment performance by reducing the cancer cells viability while providing minimal or no damage to the non-cancer cells can have a special role in the cancer chemotherapeutics as a majority of anticancer drugs provide significant damage to the other non-target sites like liver, skin, hair, nail etc. In addition, we observed that the apoptotic pathway is accompanied by the release of caspases and this programmed cell death is of at most importance in the therapeutics where the loss of apoptosis or non-release of caspases makes the cancer cells to survive for longer periods. However, in our case under the tested conditions, the continuous maintenance of apoptotic mechanism and caspase release revealed that compound **5e** is persistent enough to bring down the cancer cells viability. Further, it can be too early to comment about the apoptosis inducing ability of spiropyrrolidine heterocyclic hybrids, as the analysis that we performed in the present study are preliminary and we can only give explanation up to a certain level for the observation of high therapeutic values for some derivatives. Also, the absence of some highly reactive/aggressive groups in the moiety can be the reason for the non-observation of necrosis mechanism and that driving us to observe the alternative mechanism like the apoptosis. The observation of programmed cell death indicates that there is a series of groups that are involved sequentially to oxidize the intracellular proteins and finally bringing the cells to lose their viability and one needs to trace those chemical pathways in order to fully understand this apoptotic pathway. Our future studies are underway to examine the specific apoptosis inducing ability of these spiropyrrolidine hybrids toward the cancer cells through by means of docking, where we also have planned to investigate the behavior toward the non-targeted cells like the healthy normal ones.

## Conclusion

In summary, some novel *N*-methylated spiroheterocyclic hybrids have been synthesized via Eschweiler-Clarke reaction. MTT assay of these spiroheterocyclic hybrids indicated that the tested compounds are offering significant loss in the viability to both the cancer cell types and the efficiency of which is decided by the incubation time. It is observed that among the derivatives tested, compound **5e** with a –OCH_3_ substituent possesses higher activity against A549 cancer cells during the first 24 h of incubation period, while for reaching that level toward the Jurkat cells it needs to be incubated for 48 h. In a similar way, the activity of derivatives toward the non-cancer cells seem to be observed at high concentrations only and that too during the 48 h exposure time. Taking advantage of the quick activity of compound **5e** toward A549 cells over a 24 h period, the role of apoptotic mechanism and associated caspases were identified to be the cause of cell death. Further, exploring these mechanisms with the incorporation of the synthesized compounds for the treatment of cancer diagnosed cells can have a significant impact toward the cancer treatment as apoptosis is the majorly operating mechanism and in that way, maximum efficiency can be achieved while simultaneously reducing the non-cancer cell associated side effects.

## Data Availability Statement

All datasets generated for this study are included in the article/Supplementary Material.

## Author Contributions

RSK conceived conception, designed the experiments, performed the synthesis, analyzed the data, and wrote the paper. AA, NA, and RRK performed the synthesis. FM performed the molecular biology studies, analyzed the data, and wrote the manuscript.

## Conflict of Interest

The authors declare that the research was conducted in the absence of any commercial or financial relationships that could be construed as a potential conflict of interest.
