# Supplementary material for: In vitro Mechanistic Exploration of Novel Spiropyrrolidine Heterocyclic Hybrids as Anticancer Agents
Source: Front Chem. 2020 Jun 3;8:465. doi: 10.3389/fchem.2020.00465 (PMC7283928; doi:10.3389/fchem.2020.00465)
Supplement: Supplementary file 1 [file Data_Sheet_1.pdf]

Supplementary data

MATERIALS AND METHODS

All nitrostyrenes were prepared according to literature procedure and all other reagents and solvents were purchased from commercial suppliers and used without further purification. Reactions were monitored by thin-layer chromatography (TLC) on silica gel. Column chromatography was done on silica gel (230-400 mesh) using hexane-ethyl acetate as eluent. Melting points were recorded using open capillary tubes and are uncorrected. The ^{1}H, ^{13}C and 2-D NMR spectra were recorded on a Jeol 500 MHz instruments in $CDCl_3$ using TMS as internal standard. Standard Jeol software was used throughout. Chemical shifts are given in parts per million (δ-scale) and the coupling constants are given in Hertz. IR spectra were recorded on a Perkin Elmer system 2000 FT IR instrument (KBr). Mass spectra were recorded on a Triple quadrupole mass spectrometer, Quattro Premier equipped with an electrospray ionization source (Z–spray) coupled with an Acquity UPLC system. Elemental analyses were performed on a Perkin Elmer 2400 Series II Elemental CHNS analyzer.

General procedure for the synthesis of 3-(aryl)-4-nitro-5-(4-hydroxybenzyl)spiro[acenaphthene-2'.2-pyrrolidin]-1'-one **4(a–f)**

A mixture of β-nitrostyrenes (1 mmol), acenaphthenequinone (1 mmol) and tyrosine (1 mmol) in methanol (10 mL) was stirred at reflux for 6 h. After completion of the reaction, as indicated by TLC, the excess solvent was evaporated under reduced pressure. The resultant solid was extracted with CH_2Cl_2 (3 x 20 mL). The product was further purified by column chromatography using hexane: ethyl acetate (3:2 v/v) as eluent.

General procedure for the synthesis of 3-(3-nitrophenyl)-4-nitro-5-(4-benzyl)spiro[acenaphthene-2'.2-pyrrolidin]-1'-one **4'**

A mixture of β-nitrostyrene 1f (1 mmol), acenaphthenequinone (1 mmol) and phenylalanine (1 mmol) in methanol (10 mL) was stirred at reflux for 2h. After completion of the reaction, as indicated by TLC, the excess solvent was evaporated under reduced pressure. The resultant solid was extracted with CH2Cl2 (3x20 mL). The product was further purified by column chromatography using hexane: ethyl acetate (3:2 v/v) as eluent.

General procedure for the synthesis of spiro-pyrrolo-tetrahydroisoquinoline-acenaphthene hybrid **6**

Paraformaldehyde (1 mmol) was added to a solution of **4'** (1 mmol) in 10 mL of dicholoromethane followed by trifluoroacetic acid (0.1 mmol). The reaction mixture was stirred overnight and then extracted with dicholoromethane, washed with water and dried over Na_2SO_4. As the TLC analysis of the reaction mixture revealed the formation of product along with some other impurities of very close Rf value which could not be separated through column chromatography.

NMR and mass spectrum of a representative compound

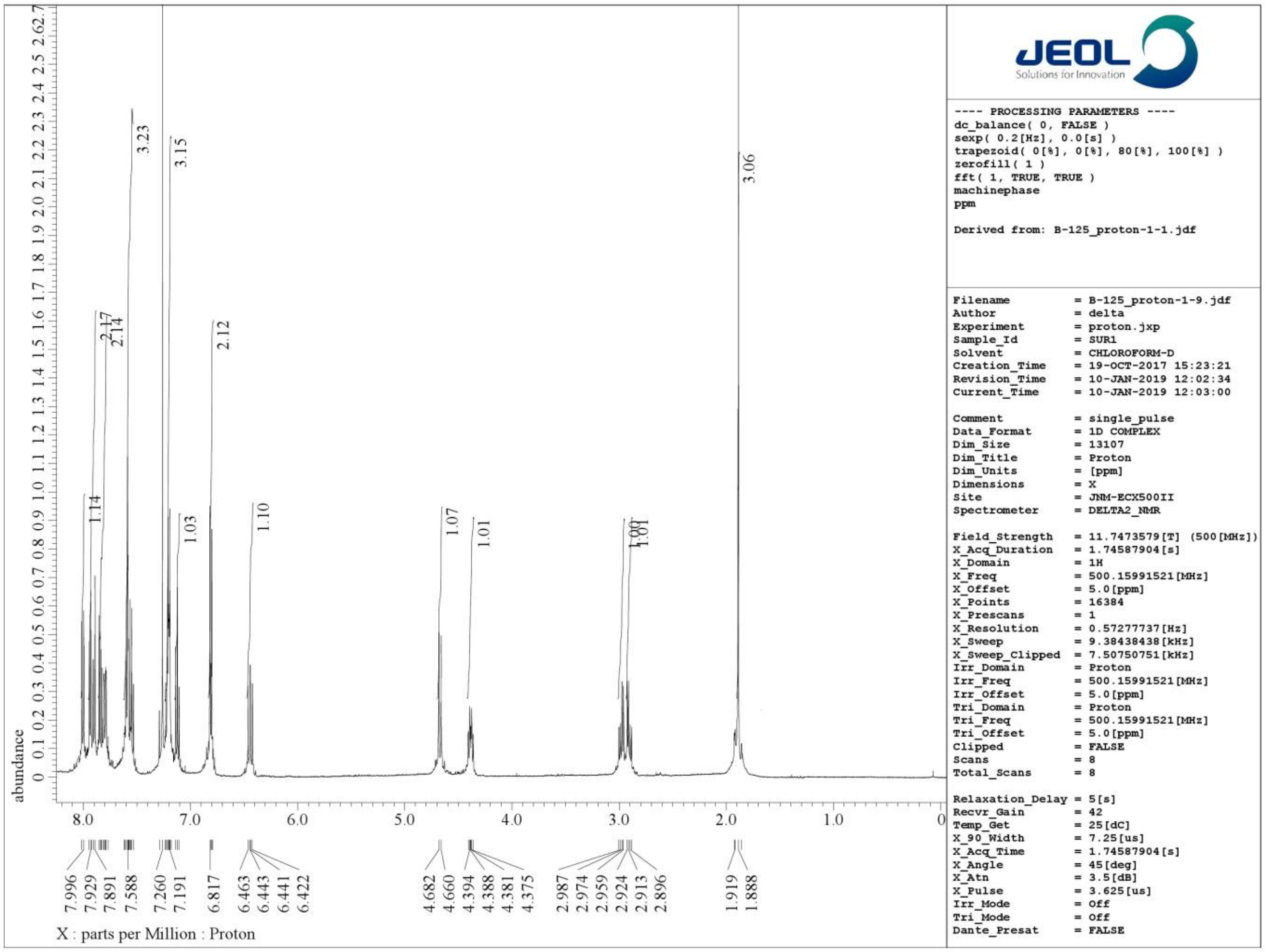

Figure S1 ^{1}H NMR spectrum of **5f**

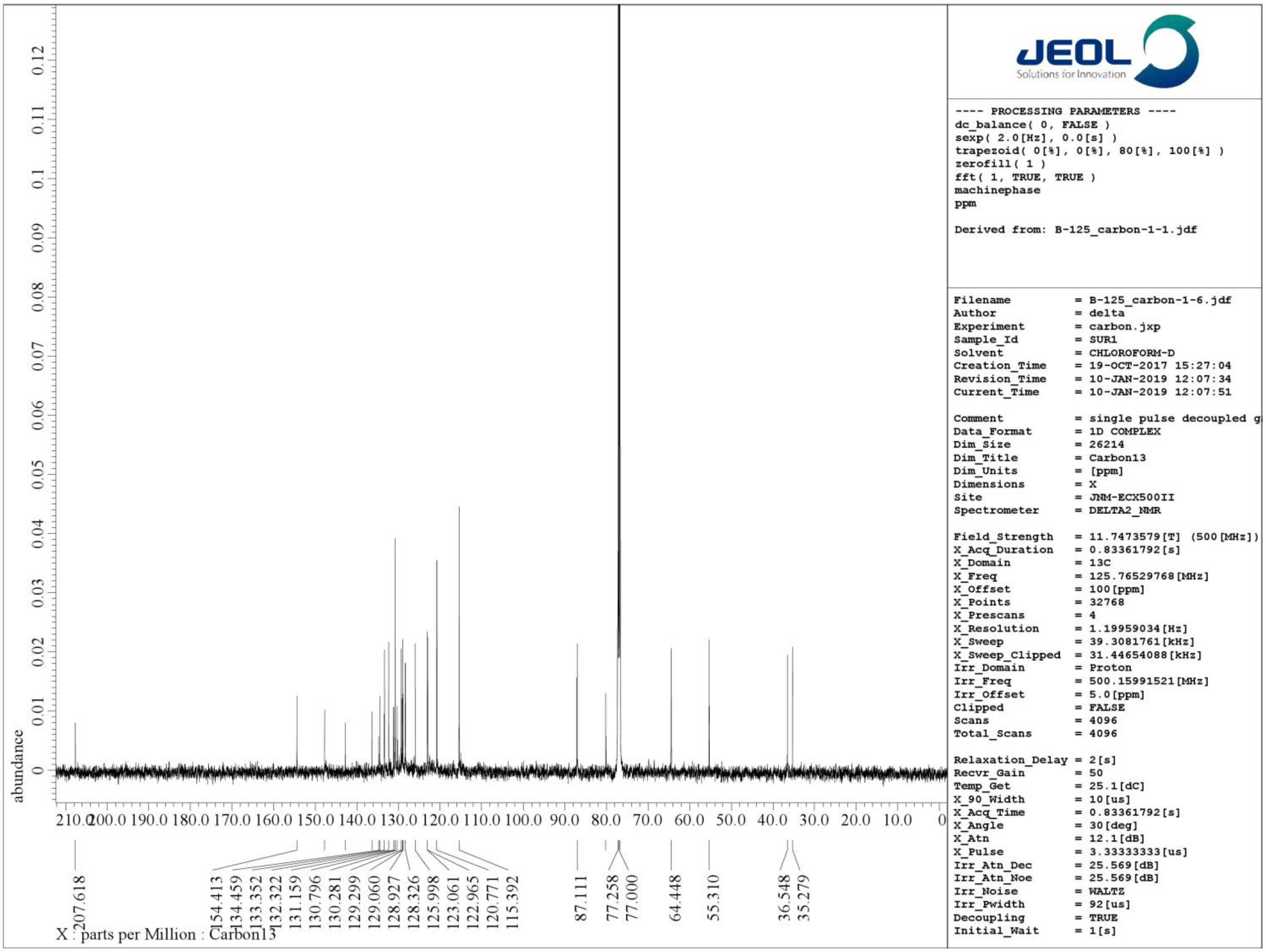

Figure S2 ^{13}C NMR spectrum of **5f**

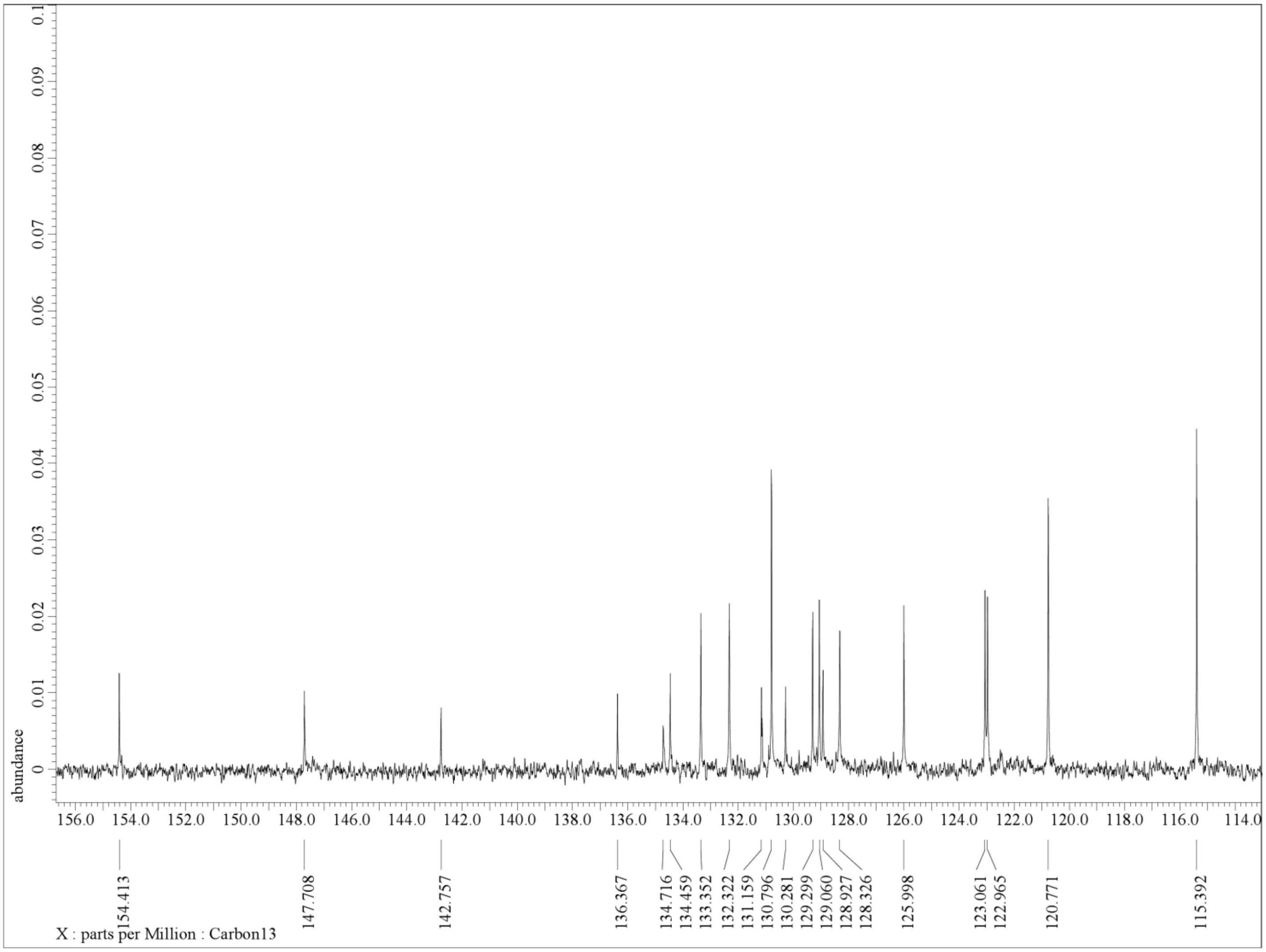

Figure S2a ^{13}C NMR spectrum of **5f**

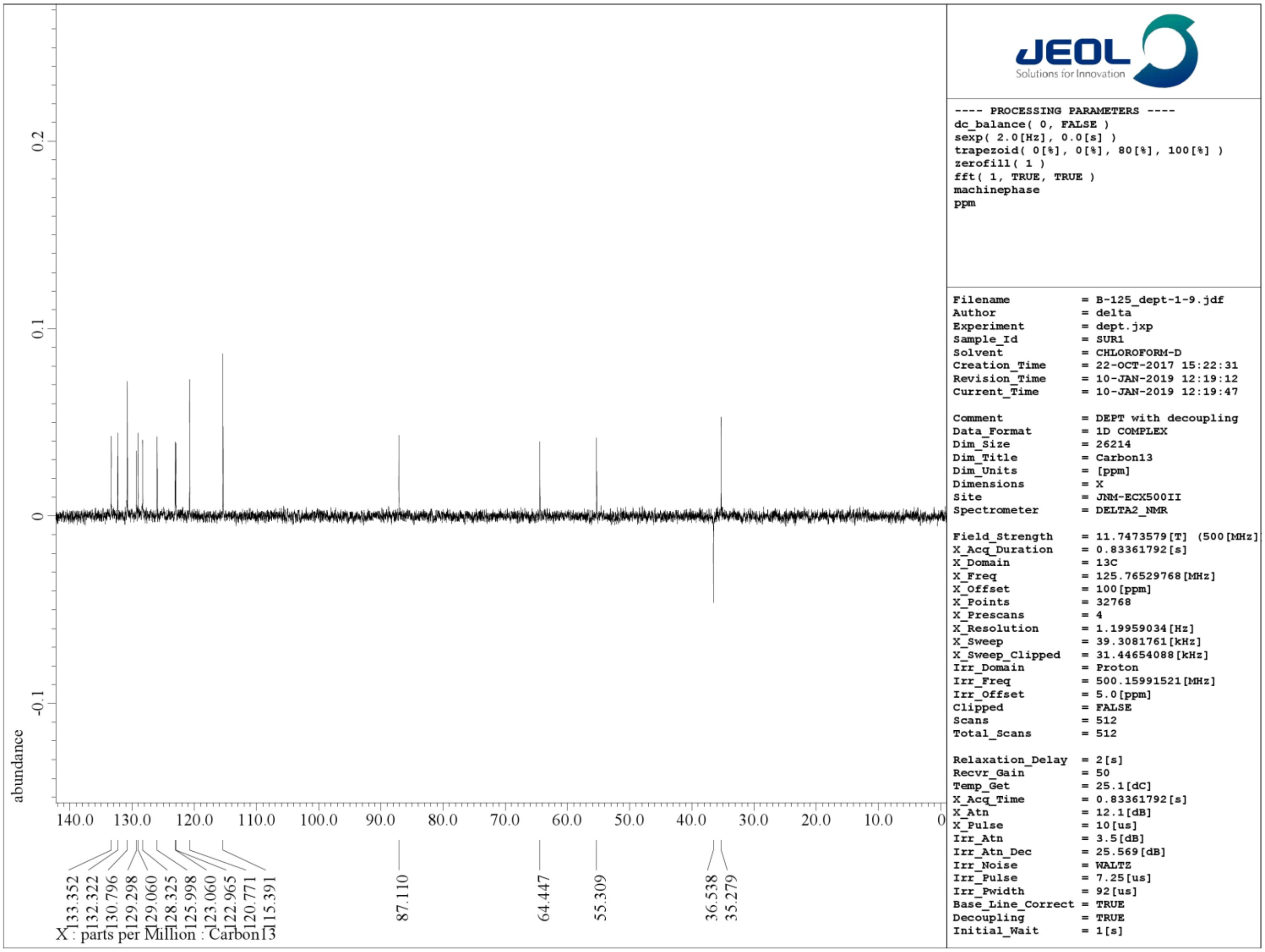

Figure S3 Dept 135 NMR spectrum of **5f**

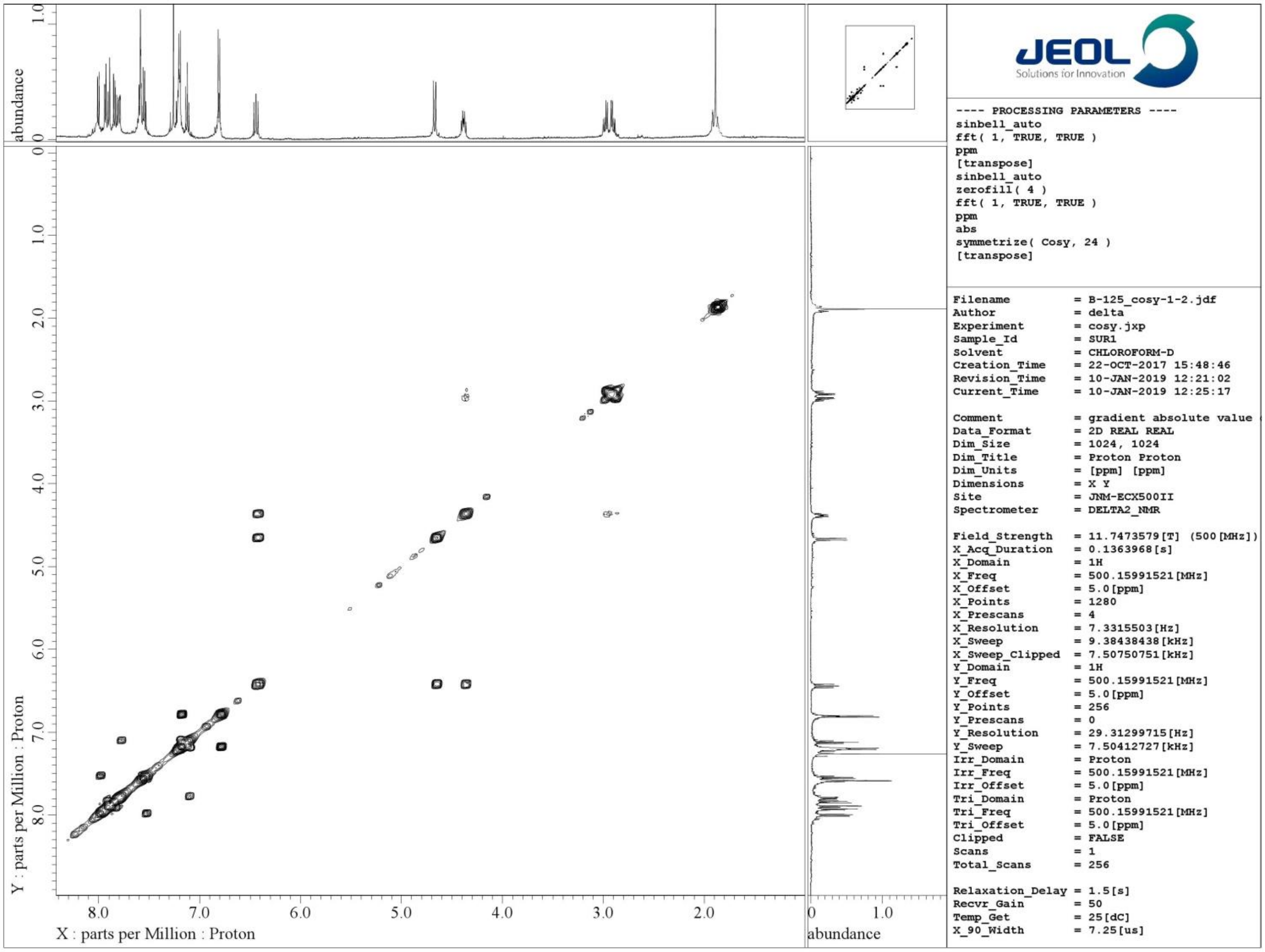

Figure S4 COSY NMR spectrum of **5f**

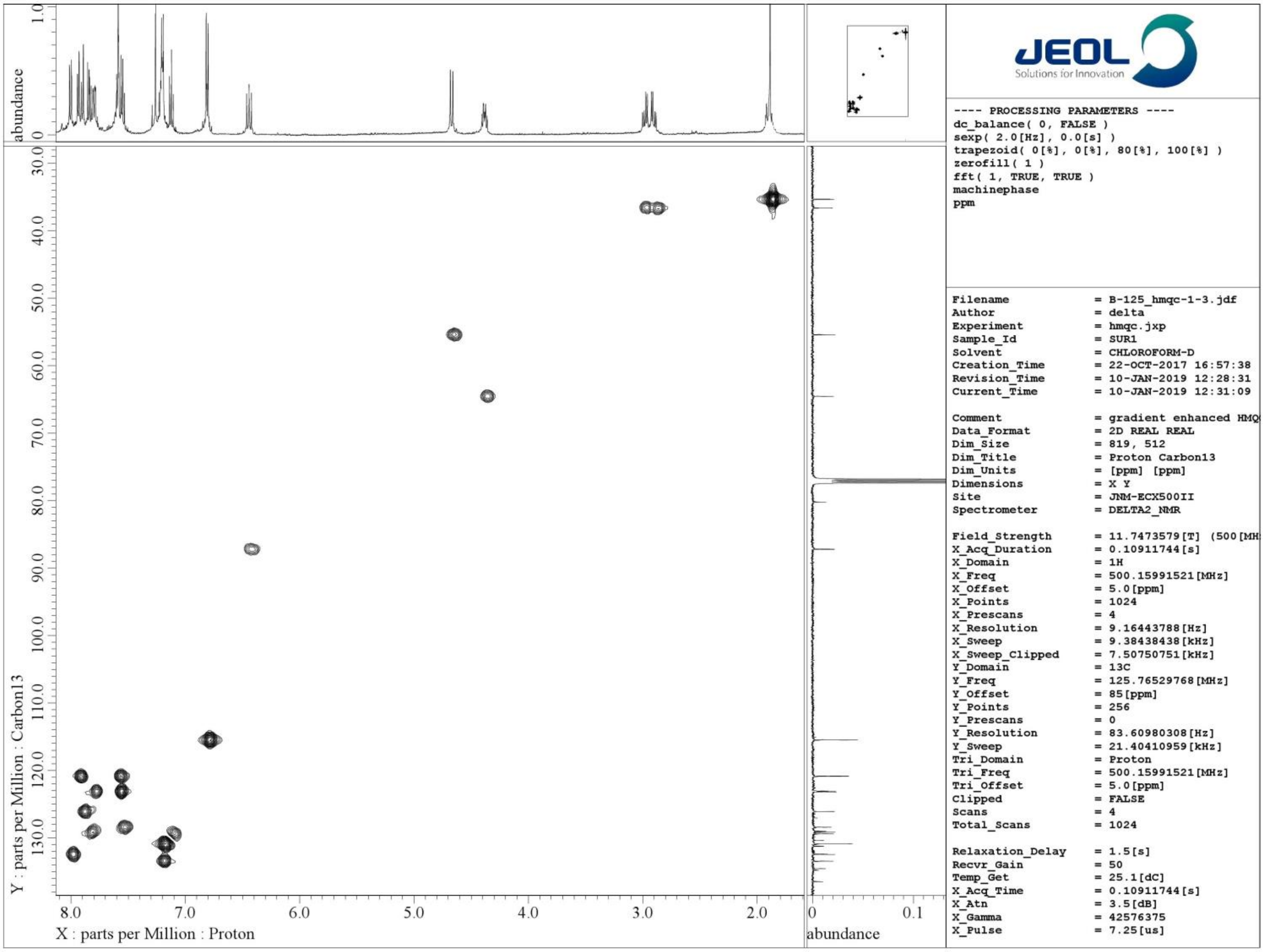

Figure S5 HMQC NMR spectrum of **5f**

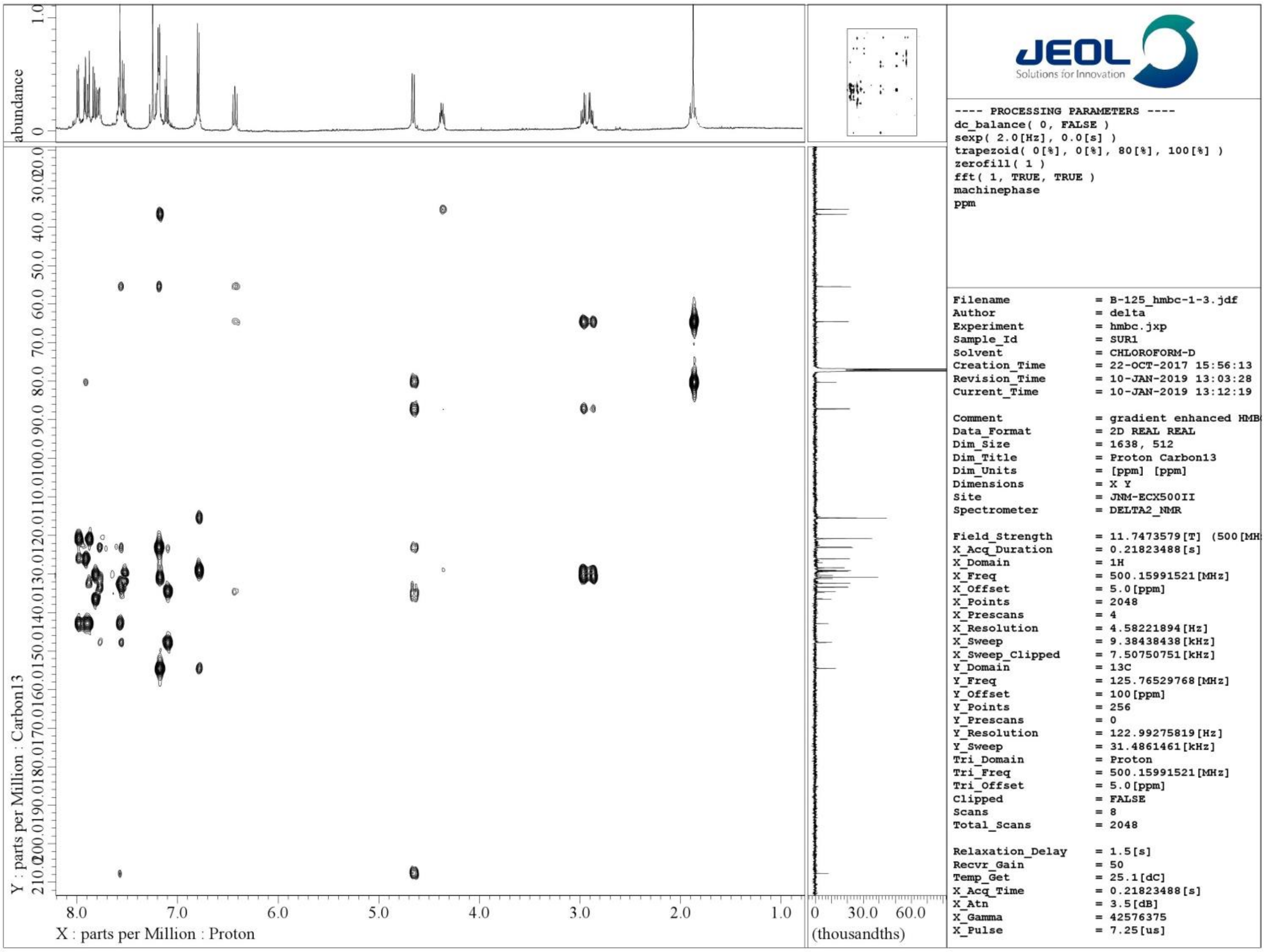

Figure S6 HMBC NMR spectrum of **5f**

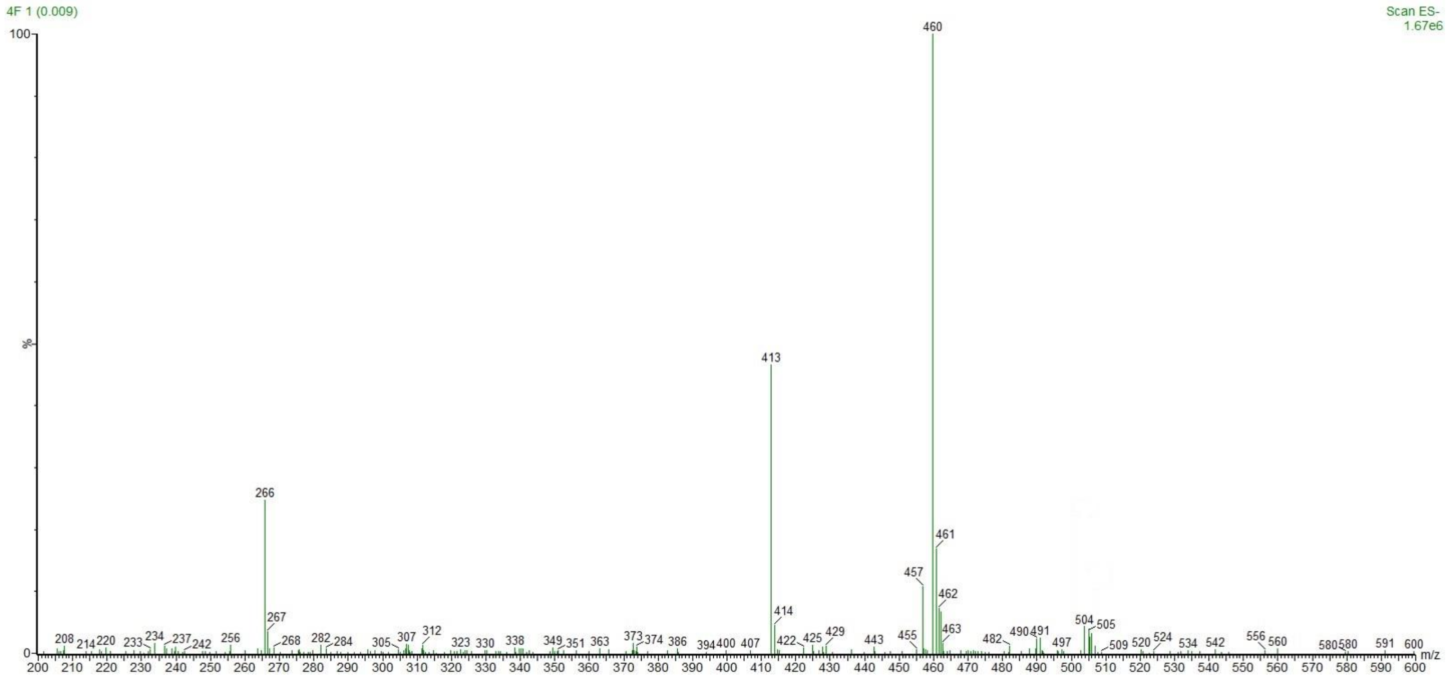

Figure S7 Mass spectrum of **5f**

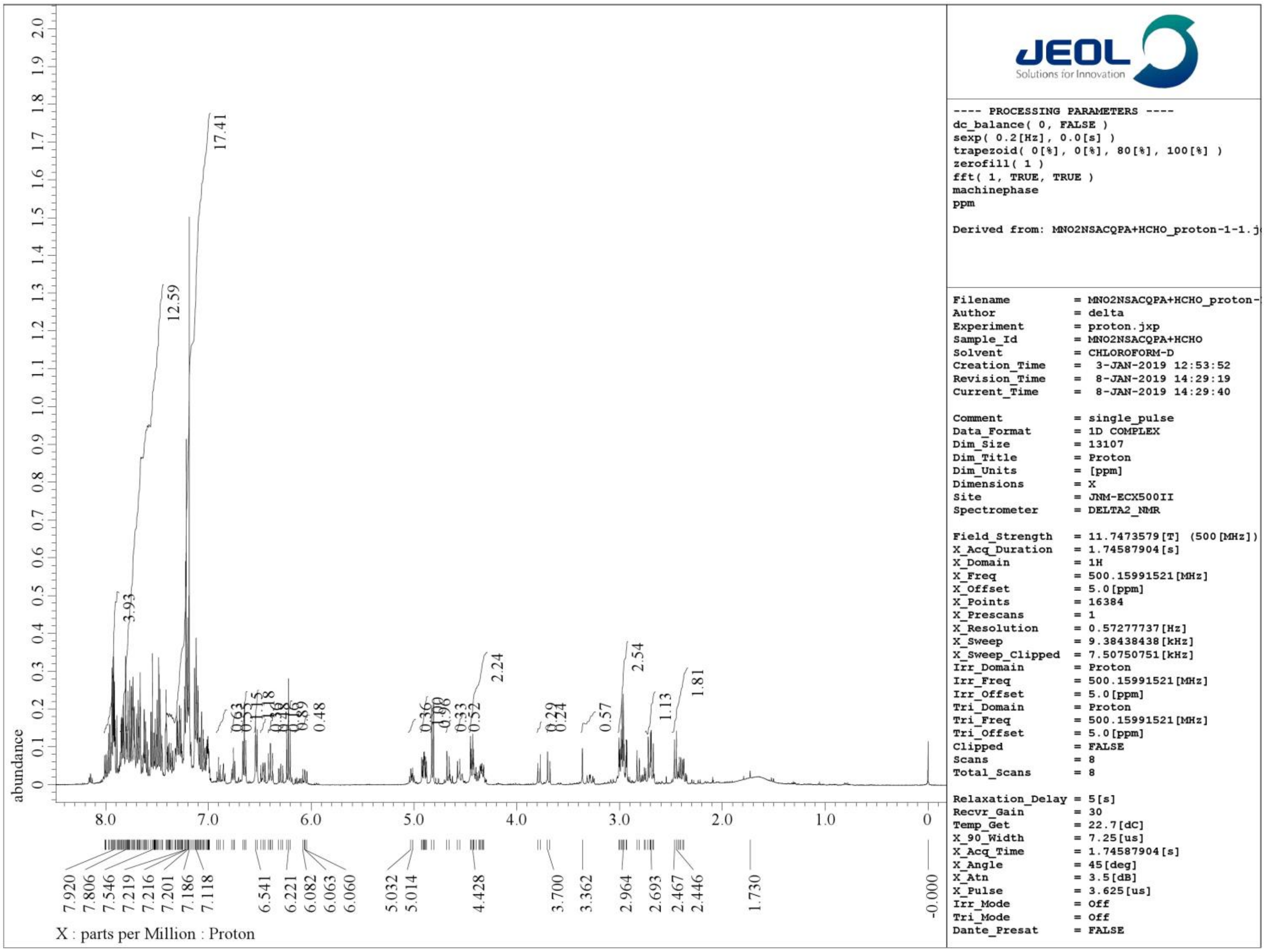

Figure S8 ^{1}H NMR spectrum of **6**

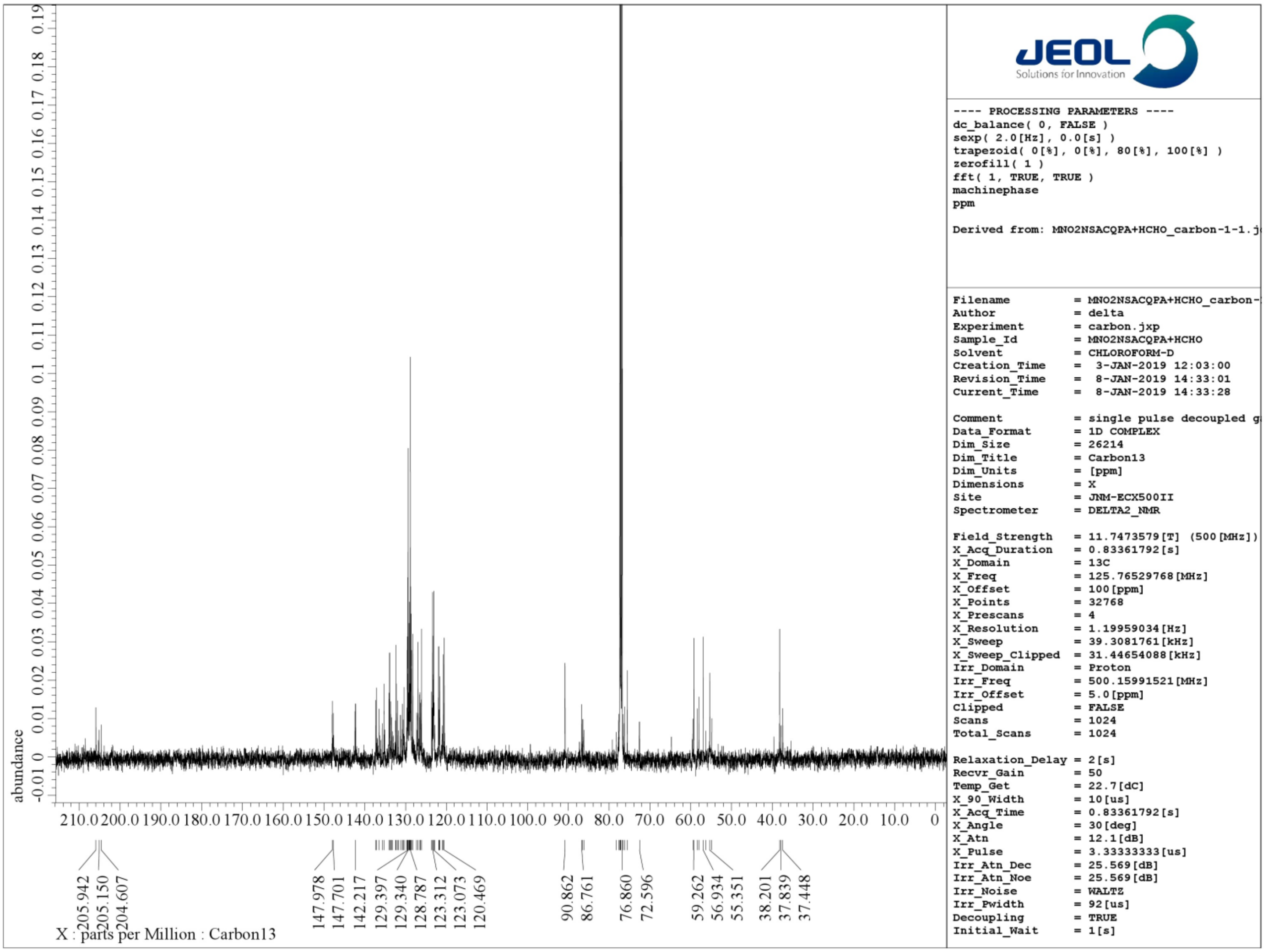

Figure S9 ^{13}C NMR spectrum of **6**

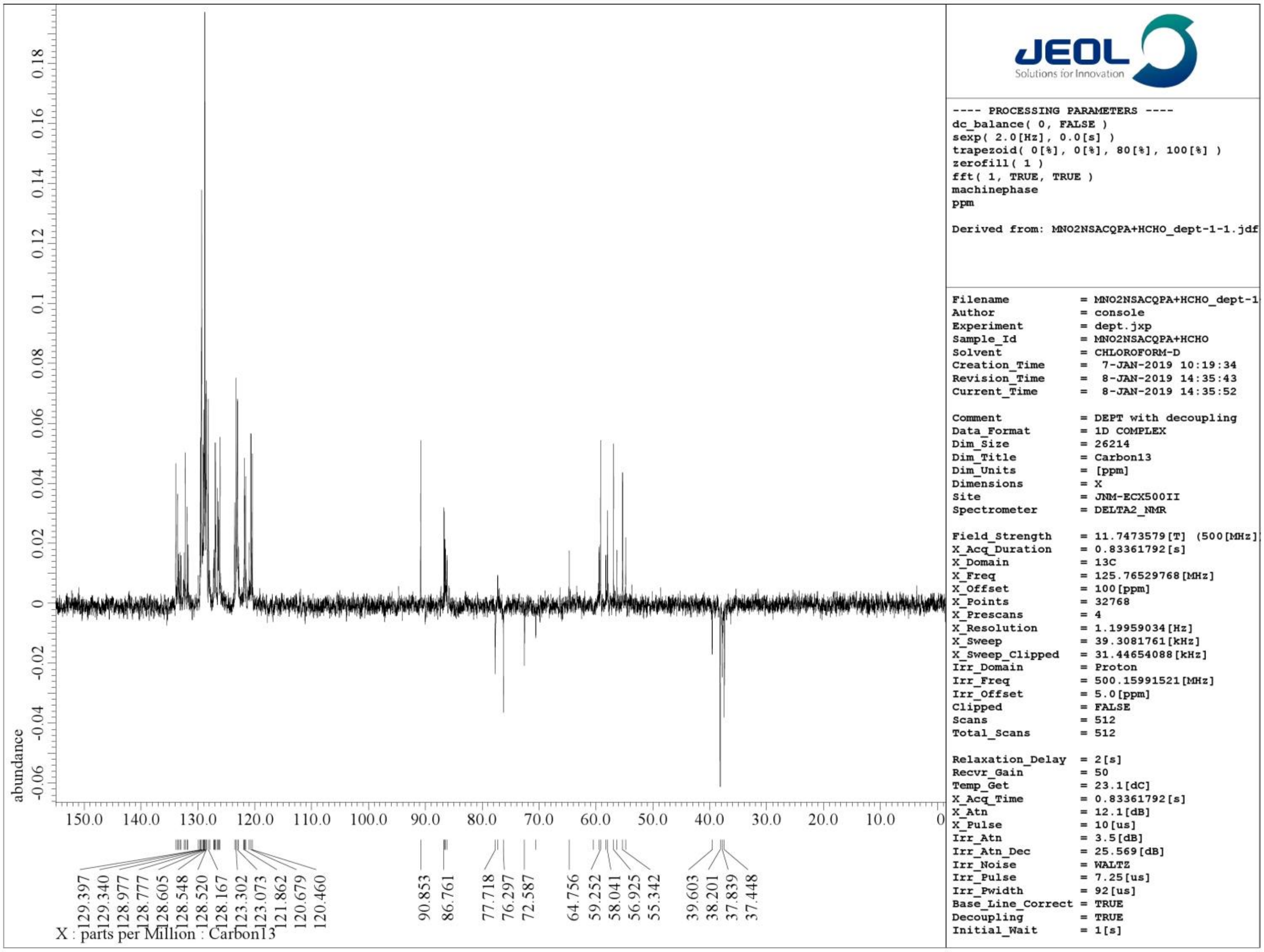

Figure S10 Dept 135 NMR spectrum of **6**

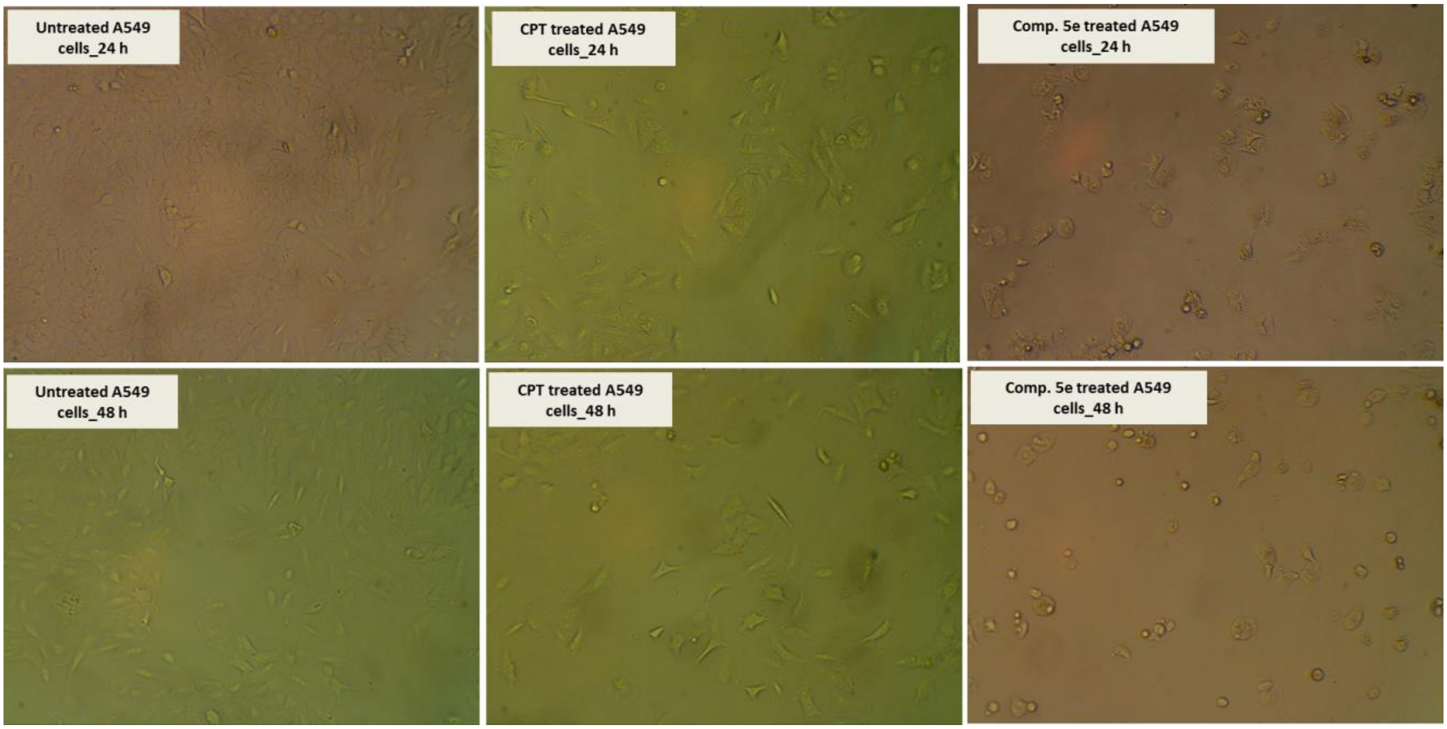

Figure S11 Comparison of normal A549 cell images during the MTT assay and following the treatment of CPT and compound **5e**.

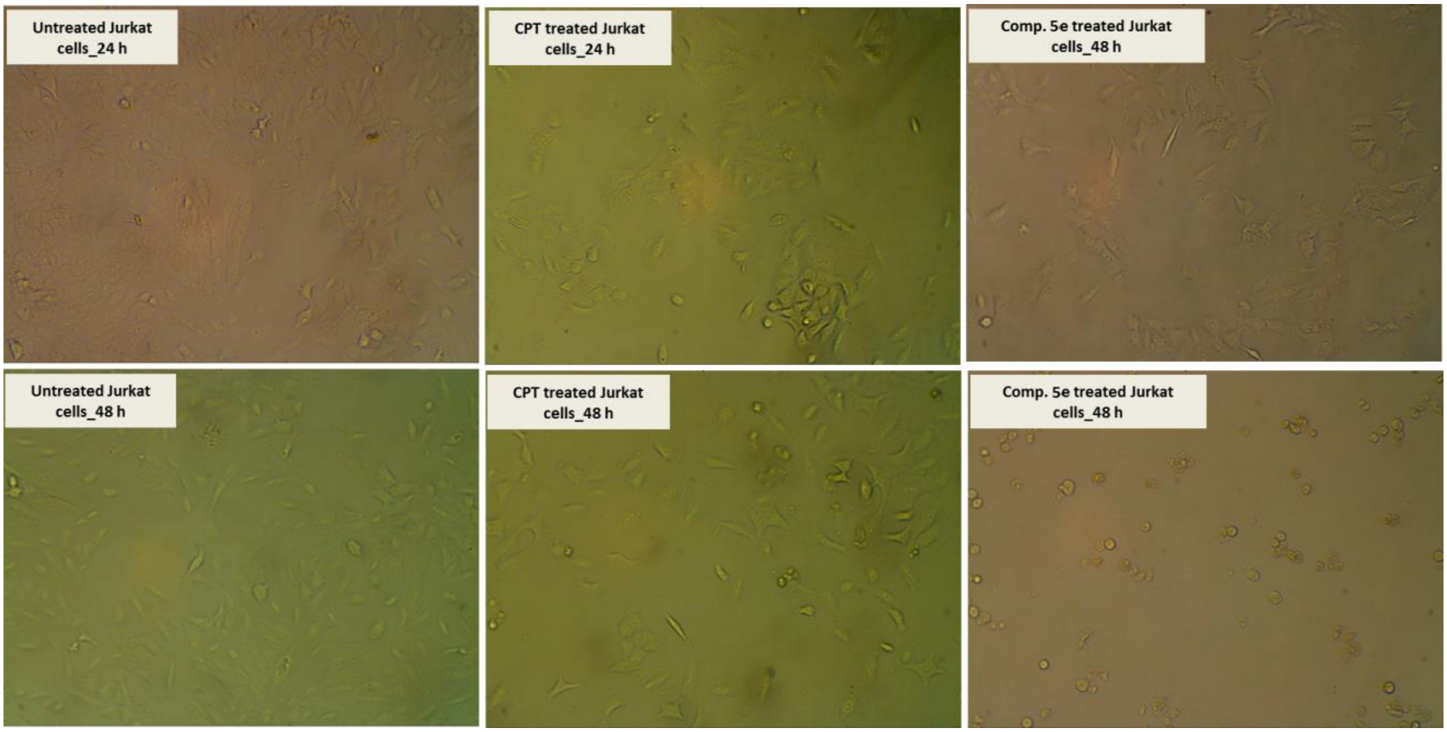

Figure S11a Comparison of normal Jurkat cell images during the MTT assay and following the treatment of CPT and compound **5e**.

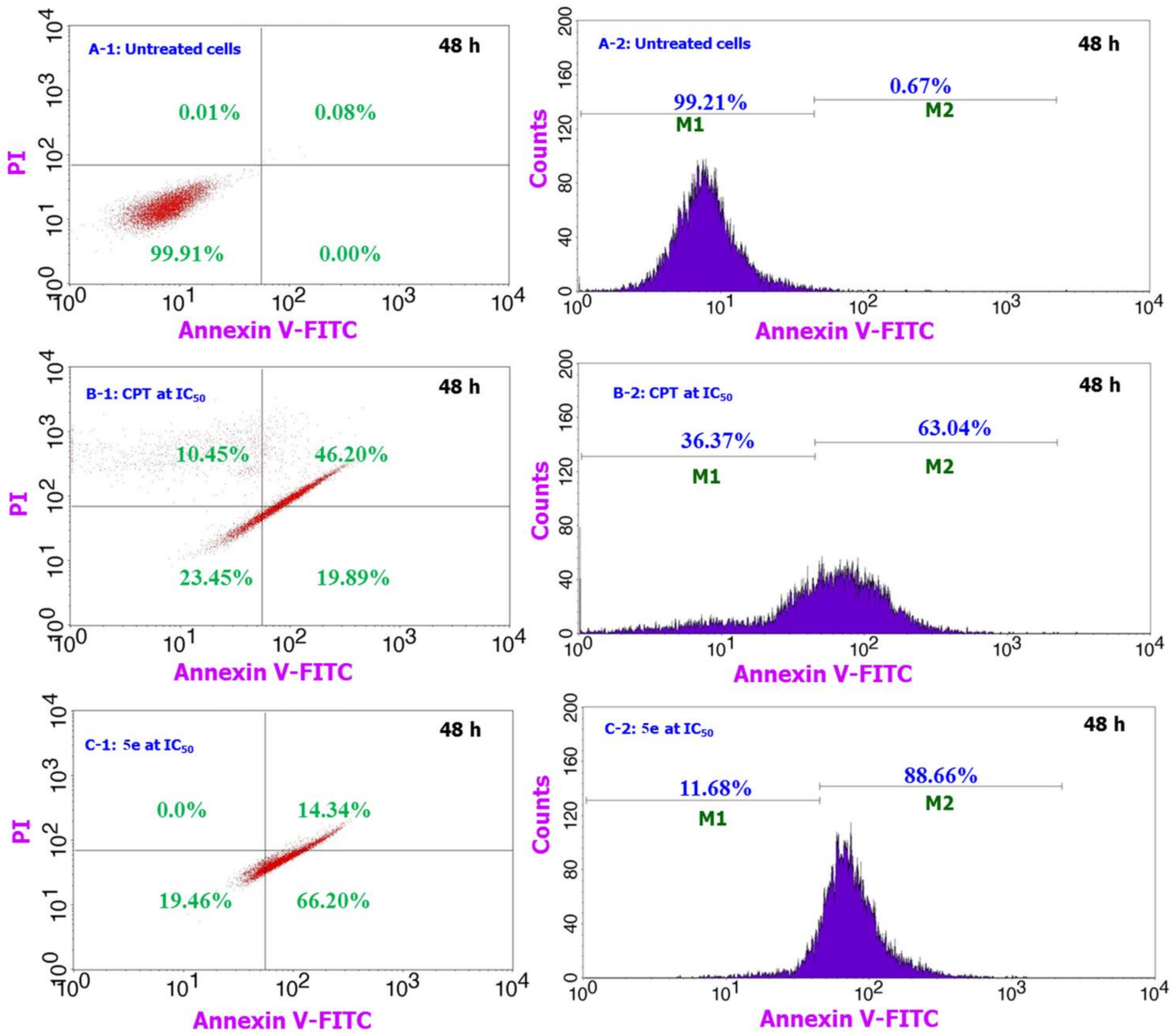

Figure S12 Apoptosis assay_48 h for the A549 cells.
